# Systematic analysis and prediction of the burden of lower respiratory tract infections attribute to non-optimal temperature, 1990–2019

**DOI:** 10.3389/fpubh.2024.1424657

**Published:** 2024-10-18

**Authors:** Yu Shi, Liping Zhang, Di Wu, Yilipa Yilihamu, Lei Wang

**Affiliations:** ^1^College of Public Health, Xinjiang Medical University, Urumqi, China; ^2^College of Medical Engineering and Technology, Xinjiang Medical University, Urumqi, China

**Keywords:** non-optimal temperatures, lower respiratory infections, disease burden, long-term trends, projections

## Abstract

**Background:**

Lower respiratory infections (LRIs) remain one of the most deadly infectious diseases in the world, and non-optimal temperature is a risk factor for LRIs. The aim of this study was to analyze the global burden of LRI attribute to non-optimal temperature and its trends from 1990 to 2019, and to project long-term trends.

**Methods:**

Excerpts from the release of the 2019 Global Burden of Disease (GBD) study, which analyses the burden of lower respiratory infections due to non-optimal temperatures from 1990 to 2019 using data on deaths and disability adjusted life years (DALYs); explores differences across regions, populations and seasons, and projects future trends in burden.

**Results:**

Between 1990 and 2019, there is a significant downward trend in the global burden of deaths and DALYs, but it remains high in infants and young children, the older adult, African countries and LOW SDI regions. Differences in geographical risk factors and economic levels lead to heterogeneous disease burdens across regions. In 2019, low SDI regions will have the highest burden, but high SDI regions will have the highest number of deaths. In addition, increasing SDI values were associated with decreasing trends in age-standardized mortality rates and disability-adjusted life years. BAPC model projections suggest a downward trend in the future burden of death and DALYs from the disease, but the improvement in the burden of death for women was not significant.

**Conclusion:**

Our study comprehensively elucidates the distribution and dynamic trends in the burden of lower respiratory tract infections due to non-optimal temperatures from 1990 to 2019 along multiple dimensions. The burden of deaths and DALYs showed an overall decreasing trend, but the improvement was uneven in different regions. In addition, the results suggest that efforts should be made to reduce lower respiratory health losses in infants, young children, and older adult populations. Effective public health policies and interventions to reduce the burden of lower respiratory tract infections should be sustained globally.

## 1 Introduction

Lower respiratory tract infections (LRIs) are among the most prevalent infectious diseases, as defined by the Global Burden of Disease (GBD) study, which categorizes them as clinician-diagnosed pneumonia or bronchiolitis. LRIs remain the deadliest infectious disease globally, ranking as the fourth leading cause of death (1, 2). They are particularly significant contributors to mortality in children under 5 years of age and adults over 70, resulting in more deaths than tuberculosis and HIV combined (3). Additionally, LRIs are associated with a range of potential complications and impose a substantial economic burden worldwide (4, 5).

The Lancet Countdown on Climate Change warns that climate change has emerged as a significant challenge to human health in the 21st century that requires urgent attention (6). Current research has extensively characterized the risk factors associated with lower respiratory infections (LRIs) (7). The burden of LRIs exacerbated by climate change, which directly impacts human health, is particularly concerning given the anticipated increase in extreme weather and climate events. Epidemiological studies have demonstrated that exposure to high temperatures poses a substantial threat to global health, with lower respiratory infections ranking among the top three health burdens linked to elevated temperatures (8). High-temperature environments influence the stability and transmission rates of respiratory viruses, thereby increasing the risk of viral transmission (9, 10). Conversely, some researchers have suggested that low-temperature environments may also elevate the disease burden associated with lower respiratory infections (11). To the best of our knowledge, fluctuations in temperature affect the activity of viruses related to these infections, which can subsequently trigger lower respiratory infections (12, 13). Cold stress, resulting from a decrease in body temperature due to the inhalation of cold air, induces a pathophysiological response. This response includes vasoconstriction of the respiratory mucosa and suppression of the immune response, both of which contribute to increased susceptibility to infection (14–16). Available data indicate that exposure to inappropriate temperatures—whether through unsuitable environmental conditions or the induction of abnormal body temperatures—heightens the risk of upper respiratory tract infections and subsequent mortality (7, 17). Therefore, both high and low temperatures may be associated with lower respiratory infections.

To date, research on the effects of high and low temperatures on lower respiratory tract infections has primarily concentrated extreme temperature exposures within individual cities. There are currently no studies that have evaluated the global burden of lower respiratory tract infections attributable to non-optimal temperatures across various regions, countries, age, gender, or socioeconomic statuses. Additionally, long-term trends in the burden of lower respiratory tract infections due to non-optimal temperatures among different age groups and sexes have not been thoroughly examined, and projections regarding these long-term trends are lacking. Assessing the patterns of the global burden of lower respiratory tract infections induced by non-optimal temperatures is crucial for informing prevention and control strategies in the context of climate change. Therefore, the aim of this study was to examine the temporal trend of the burden of non-optimal temperature-induced lower respiratory tract infections from 1990 to 2019 and the effect of age, period and cohort on it using data from GBD 2019, and to further predict the disease burden in the next decade.

## 2 Methods

### 2.1 Data source and data collection

The Global Burden of Disease (GBD) represents the most comprehensive epidemiological study conducted to date, providing a powerful resource for researchers to quantify the global health toll of hundreds of diseases, injuries and risk factors. It has epidemiological data on more than 350 diseases and injuries by sex and age for 195 countries between 1990 and 2019 (1). All anonymized data are publicly accessible on the Institute for Health Metrics and Evaluation (IHME) website and can be found online at http://ghdx.healthdata.org/gbd-results-tool. The informed consent form was reviewed and approved by the University of Washington Institutional Review Board.

For the Global Burden of Disease (GBD) 2019 study, the case definition of LRIs was clinician-diagnosed pneumonia or bronchiectasis. The International Classification of Diseases (ICD) 9th edition codes were 073.0, 073.6, 079.82, 466–469, 480–489, 513.0, and 770.0, and ICD 10th edition codes were coded as codes A48.1, J09-J22, J85.1, P23-P23.9, and U04. Mean daily temperatures at each location were obtained from the European Medium-Range Weather Forecasting Center. The Theoretical Minimum Temperature Risk Exposure Level (TMREL) is defined as the temperature estimated to be associated with the lowest risk of death from all included causes for a given location and year. High temperature exposure is defined as exposure to temperatures above the TMREL, and low temperatures are defined as temperatures below the TMREL (18, 19). We extracted estimates of deaths from lower respiratory infections due to unsuitable temperatures, disability-adjusted life-years (DALYs), and the corresponding age-standardized rates for the years 1990–2019 and their 95% uncertainty intervals (UIs) from the GBD 2019. The Sociodemographic Index (SDI) combines mean income, mean education level and total fertility rate to produce a comprehensive profile of the country’s health-related socioeconomic level, with higher SDIs implying better socioeconomic development: according to the SDI, regions are classified into five categories, including low (<0.46), low-middle (0.46–0.60), medium (0.61–0.69), medium-high (0.70–0.81), and high (>0.81) (20).

### 2.2 Statistical analysis

ASMR and ASDR are reported per 100,000 individuals or person-years, accompanied by a 95% uncertainty interval (UI). The average annual percent change (AAPC) for ASMR and ASDR was calculated using Joinpoint regression software. Time-trend changes in the burden of lower respiratory tract infections due to non-optimal temperatures were estimated from 1990 to 2019. The Joinpoint model is a segmented regression based on the temporal characteristics of the disease distribution, using multiple joinpoints, dividing the study period into intervals, and fitting and optimizing the trend for each interval. This approach addresses the limitations of the traditional modeling method of capturing local changes (21). An average percent change (APC) or AAPC greater than 0 is defined as an upward trend, and vice versa (22). This analysis was conducted using Joinpoint version 5.0.2 regression program software.

Age-Period-Cohort (APC) analysis is widely utilized in sociology and epidemiology. APC models, which are based on the Poisson distribution, can effectively reflect the temporal trends of morbidity or mortality by age, period and cohort (23, 24). To address the problem of non-identifiability due to the linear relationship between age, period, and cohort, some researchers have proposed the intrinsic estimator (IE) approach associated with the APC model. This method allows for the decomposition of the three temporal trends and to provide unbiased and relatively efficient estimates (25, 26). In the APC model using the IE approach, the age-specific rates are appropriately recoded into consecutive 5-year age groups (<5, 5–9, 10–14..., 90–94, >95 years), consecutive 5-year periods (1990–2019), and corresponding consecutive 5-year birth cohorts (1985–1989, 1900–1904..., 2010–2014, 2015–2019) This categorization facilitates the estimation of age, period, and cohort effects on mortality from lower respiratory tract infections and disability-adjusted life years (DALYs). The general expression for the APC model is:


$$Yi=\mu +\alpha age_{i}+\mathrm{\beta perio}d_{i}+\mathrm{\gamma cohor}t_{i}+\varepsilon$$

where Y represents the dependent variable, *μ* represents the intercept, *α*, *β*, and *γ* are the effect coefficients for age, period, and cohort, respectively, and *ε* is the random error, age = period - cohort. The R-based APC web tool^1^ was used for solving identifiable problems, estimating parameters and associated statistical hypothesis testing.

In addition, a Bayesian age-period-cohort analysis (BAPC) model was used to predict overall trends in the global burden of lower respiratory tract infections from 2020 to 2030 and trends by age stratification. The core of the BAPC model lies in its Bayesian framework. Unlike traditional frequentist methods, Bayesian approaches use prior knowledge and observational data to update parameter estimates, offering greater flexibility in the face of uncertainty. By simultaneously considering age, period, and cohort effects, the BAPC model enhances our understanding of the complex dynamics of disease occurrence. It excels in probabilistic forecasting, especially when well-calibrated, resulting in narrower prediction intervals. The model assumes that the influences of adjacent cohorts in age, period, and time are similar, with classifications aligning with those of traditional APC models. Comprehensive visualization of the BAPC model projections was performed based on R version 4.3.2.

## 3 Results

### 3.1 Burden of non-optimal temperature-induced lower respiratory tract infections and changing trends

**Figure 1:**
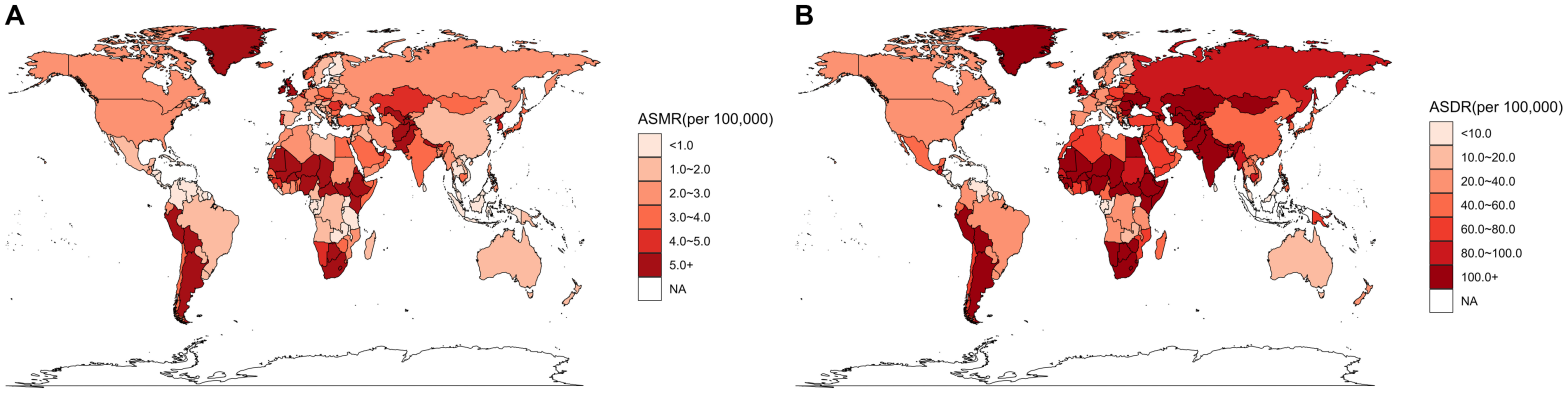
Spatial distribution of LRIs due to non-optimal temperatures in 2019, **(A)** for ASMR and **(B)** for ASDR.

Non-optimal temperature-induced lower respiratory tract infection burden and trends in 2019 are displayed in Table 1, Figure 1, and Supplementary Figure S1. Globally the number of deaths from lower respiratory infections due to non-optimal temperatures decreased to 245.814 (174.760, 342.302) from 359.594 (257.631, 608.180) in 1990, with the ASMR showing a significant downward trend 7.671 (5.647, 12.098) per 100,000 to 3.411 (2.421, 4.775) with an AAPC of −2.839 (−3.096, −2.581). Both DALY and ASDR also showed a downward trend, with ASDR decreasing from 383.147 (268.486, 685.095) to 121.467 (76.078, 203.233) and an AAPC of −3.883 (−4.341, −3.423). After eliminating the differences in population and age structure of the countries, Lesotho, Nigeria, Chad, Argentina, and Afghanistan had the highest ASMR for LRIs attributed to non-optimal temperatures, and Lesotho, Nigeria, Nigeria, Chad, and Tajikistan had the highest ASDR.

**Table 1:** Number and age-standardized rates of deaths (A), DALYs (B) for LRIs attributable to non-optimal temperature in 1990 and 2019, and corresponding temporal trends from 1990 to 2019.

A-Deaths	1990		2019		1990–2019	
Location	Deaths Number*10^3^	ASMR per 100,000	Deaths Number*10^3^	ASMR per 100,000	AAPC	*p*
Global	359.594(257.631,608.18)	7.671(5.647,12.098)	245.814(174.76,342.302)	3.411(2.421,4.775)	−2.839(−3.096,-2.581)	<0.001
Sex						
Male	186.247(132.370,318.098)	8.698(6.394,13.172)	127.172(89.825,177.054)	4.038(2.917,5.553)	−2.681(−2.930,-2.431)	<0.001
Female	173.346(125.125,291.736)	6.976(5.093,11.157)	118.641(84.149,165.701)	2.094(2.976,4.304)	−2.980(−3.242,-2.718)	<0.001
SDI						
High SDI	51.959(38.855,67.443)	5.157(3.857,6.699)	69.216(51.479,88.585)	3.025(2.275,3.845)	−1.950(−2.185,-1.715)	<0.001
High-middle SDI	47.530(34.783,62.225)	5.095(3.748,6.635)	41.017(28.315,55.519)	2.272(1.583,3.064)	−2.812(−3.127,-2.495)	<0.001
Middle SDI	91.968(69.51,133.785)	6.354(4.751,9.428)	38.665(25.473,57.769)	2.007(1.325,2.999)	−3.910(−4.243,-3.576)	<0.001
Low-middle SDI	99.923(64.883,200.881)	8.709(5.564,17.865)	50.472(31.163,76.884)	3.853(2.372,5.865)	−3.031(−3.183,-2.879)	<0.001
Low SDI	68.121(36.346,167.242)	11.075(6.0144,27.743)	46.374(22.767,97.094)	5.182(2.672,10.288)	−2.687(−2.978,-2.395)	<0.001
GBD region						
Andean Latin America	4.377(3.011,6.046)	13.619(9.369,19.051)	2.939(1.769,4.31)	5.418(3.277,7.924)	−2.891(−3.387,-2.392)	<0.001
Australasia	0.454(0.35,0.574)	2.256(1.723,2.863)	0.727(0.538,0.92)	1.252(0.935,1.582)	−2.030(−3.156,-0.892)	<0.001
Caribbean	0.360(0.056,1.301)	1.206(0.236,4.203)	0.201(−0.105,0.571)	0.397(−0.236,1.16)	−2.672(−3.305,-2.034)	<0.001
Central Asia	10.838(8.102,14.777)	12.788(9.552,17.519)	4.598(3.288,6.288)	5.843(4.161,7.974)	−2.612(−2.978,-2.245)	<0.001
Central Europe	7.422(5.361,9.992)	6.468(4.654,8.709)	6.449(4.439,8.865)	3.258(2.237,4.471)	−2.405(−3.561,-1.236)	<0.001
Central Latin America	4.427(2.908,7.286)	3.465(2.267,5.838)	2.683(1.495,4.276)	1.187(0.661,1.896)	−3.601(−4.107,-3.090)	<0.001
Central Sub-Saharan Africa	2.176(−0.093,5.589)	4.372(−0.218,11.087)	0.993(−0.87,2.982)	1.513(−1.46,4.754)	−3.817(−5.663,-1.936)	<0.001
East Asia	85.444(62.505,111.724)	9.186(6.825,12.012)	25.612(17.005,35.313)	1.807(1.227,2.448)	−5.582(−6.343,-4.185)	<0.001
Eastern Europe	5.838(1.141,10.134)	2.834(0.599,4.902)	7.578(0.645,13.604)	2.640(0.259,4.699)	−0.172(−1.030,0.694)	0.696
Eastern Sub-Saharan Africa	17.862(8.313,35.036)	9.765(4.599,18.829)	7.271(1.154,17.221)	3.431(0.723,7.365)	−3.403(−3.79,-3.014)	<0.001
High-income Asia Pacific	11.425(9.234,13.794)	7.006(5.606,8.504)	23.024(17.117,28.747)	3.684(2.805,4.573)	−2.330(−3.222,-1.43)	<0.001
High-income North America	13.525(10.111,17.5)	3.722(2.787,4.809)	15.184(11.014,19.648)	2.186(1.61,2.808)	−2.020(−2.459,-1.578)	<0.001
North Africa and Middle East	25.994(18.353,36.11)	7.315(5.383,9.719)	14.218(10.175,18.506)	3.546(2.568,4.564)	−2.517(−2.918,-2.114)	<0.001
Oceania	0.201(0.071,0.514)	3.198(1.118,8.872)	0.143(−0.011,0.338)	1.180(−0.135,2.981)	−3.137(−3.992,-2.275)	<0.001
South Asia	91.324(52.106,178.886)	8.284(4.734,17.148)	53.964(27.225,82.24)	4.087(2.065,6.305)	−2.778(−2.99,-2.565)	<0.001
Southeast Asia	11.019(1.209,52.336)	2.779(0.244,13.730)	4.487(−1.344,20.98)	0.926(−0.267,4.288)	−3.816(−4.681,-2.944)	<0.001
Southern Latin America	3.066(2.024,4.483)	7.374(4.888,10.798)	7.154(4.665,10.122)	8.437(5.507,11.923)	0.479(−0.348,1.313)	0.257
Southern Sub-Saharan Africa	4.184(3.14,5.416)	10.424(7.822,13.370)	3.598(2.386,4.942)	6.605(4.493,8.969)	−1.379(−1.703,-1.053)	<0.001
Tropical Latin America	2.855(0.225,9.913)	2.660(0.263,8.280)	2.611(0.247,6.963)	1.179(0.112,3.173)	−2.561(−3.312,-1.804)	<0.001
Western Europe	28.079(19.829,37.669)	5.008(3.5358,6.731)	33.895(23.618,45.165)	2.924(2.045,3.889)	−1.964(−2.358,-1.568)	<0.001
Western Sub-Saharan Africa	28.726(7.142,111.17)	12.399(2.946,53.067)	28.485(8.185,86.43)	7.106(1.852,23.056)	−1.654(−2.033,-1.273)	<0.001

B-DALYs	1990		2019			
Location	DALYs Number*10^5^	ASDR per 100,000	DALYs Number*10^5^	ASDR per 100,000	AAPC	P
Global	229.591(159.591,417.311)	383.147(268.486,685.095)	85.108(53.865,141.787)	121.467(76.078,203.233)	−3.883(−4.341,-3.423)	<0.001
Sex						
Male	121.033(84.357,217.314)	403.938(283.240,709.729)	44.984(29.053,74.322)	129.727(84.238,214.046)	−4.079(−4.185,-3.974)	<0.001
Female	108.558(75.281,198.417)	365.519(255.096,661.150)	40.124(25.489,66.023)	72.103(115.127,193.093)	−4.118(−4.226,-4.010)	<0.001
SDI						
High SDI	8.223(6.227,10.563)	86.648(65.666,111.129)	8.365 (6.374,10.568)	44.959(34.543,56.527)	−2.373(−2.611,-2.134)	<0.001
High-middle SDI	25.620(18.697,33.889)	251.057(183.208,331.394)	8.395 (5.408,11.749)	53.786(34.847,75.260)	−5.221(−5.413,-5.029)	<0.001
Middle SDI	66.599(50.273,97.118)	348.713(263.413,506.271)	13.077 (8.940,20.255)	65.718(44.820,102.559)	−5.573(−5.894,-5.251)	<0.001
Low-middle SDI	75.912(49.565,149.883)	485.466(315.895,974.443)	23.466 (14.367,36.306)	147.352(90.465,225.799)	−4.15(−5.084,-3.207)	<0.001
Low SDI	53.183(28.408,130.000)	603.286(322.322,1482.184)	31.784 (15.110,67.858)	224.414(109.534,472.228)	−5.573(−5.894,-5.251)	<0.001
GBD region						
Andean Latin America	2.831(1.919,3.940)	593.946(404.863,827.351)	0.752 (0.440,1.089)	127.310(74.885,183.979)	−4.949(−5.247,-4.650)	<0.001
Australasia	0.077(0.06,0.096)	37.822(29.807,47.390)	0.082 (0.062,0.104)	17.401(13.297,21.973)	−2.763(−3.846,-1.667)	<0.001
Caribbean	0.191(0.015,0.713)	49.973(5.418,184.507)	0.047(−0.056,0.189)	10.352(−13.636,43.349)	−3.805(−4.468,-3.137)	<0.001
Central Asia	8.827(6.605,12.074)	966.789(723.243,1319.343)	2.829 (2.032,3.883)	312.920(224.459,429.249)	−3.883(−4.177,-3.588)	<0.001
Central Europe	2.817(2.019,3.782)	276.079(197.716,369.950)	1.244 (0.856,1.706)	83.279(58.132,114.406)	−3.995(−5.177,-2.797)	<0.001
Central Latin America	2.829(1.846,4.499)	145.852(95.553,237.831)	0.823 (0.447,1.346)	35.701(19.275,58.634)	−4.767(−4.989,-4.545)	<0.001
Central Sub-Saharan Africa	1.597(−0.066,4.154)	186.293(−8.023,470.307)	0.533(−0.383,1.586)	44.153(−39.095,133.971)	−5.093(−6.710,-3.449)	<0.001
East Asia	60.942(43.518,80.914)	525.834(376.697,695.499)	5.669 (3.591,7.751)	47.092(30.315,64.108)	−8.017(−8.617,-7.413)	<0.001
Eastern Europe	2.583(0.538,4.508)	139.7318(31.0337,242.001)	2.388 (0.243,4.235)	97.830(11.560,171.458)	−1.109(−1.883,-0.328)	0.0054
Eastern Sub-Saharan Africa	13.233(6.036,26.31)	449.416(212.342,876.209)	3.999 (0.494,9.933)	100.166(15.715,236.841)	−4.826(−5.202,-4.449)	<0.001
High-income Asia Pacific	1.872(1.514,2.268)	110.975(89.450,134.543)	2.479 (1.911,3.058)	49.723(39.516,61.218)	−2.845(−3.758,-1.924)	<0.001
High-income North America	2.222(1.675,2.845)	66.751(50.297,85.031)	2.161 (1.639,2.733)	38.070(28.880,47.952)	−1.990(−2.546,-1.430)	<0.001
North Africa and Middle East	19.847(13.795,28.356)	405.527(285.256,566.679)	6.044 (4.145,8.039)	114.646(79.965,151.293)	−4.328(−4.603,-4.052)	<0.001
Oceania	0.153(0.055,0.38)	172.786(60.954,442.718)	0.105(−0.006,0.243)	62.076(−3.48,145.269)	−3.094(−3.725,-2.459)	<0.001
South Asia	70.320(40.516,136.521)	469.782(270.478,909.309)	26.673(13.211,41.393)	171.283(84.593,266.711)	−3.630(−3.850,-3.410)	<0.001
Southeast Asia	7.788(0.923,36.593)	144.896(16.825,687.024)	1.455(−0.415,7.105)	26.283(−7.527,127.560)	−5.799(−6.568,-5.024)	<0.001
Southern Latin America	0.896(0.593,1.306)	190.102(125.648,276.880)	1.157 (0.768,1.619)	147.564(97.012,205.694)	−0.764(−1.182,-0.345)	<0.001
Southern Sub-Saharan Africa	2.516(1.838,3.278)	442.602(330.572,572.598)	1.404 (0.876,1.996)	201.204(127.457,284.522)	−2.808(−3.356,-2.256)	<0.001
Tropical Latin America	1.710(0.101,6.666)	112.919(6.401,423.060)	0.571 (0.028,1.666)	26.741(0.466,79.869)	−4.724(−5.449,-3.994)	<0.001
Western Europe	4.003(2.843,5.356)	77.490(55.222,103.377)	3.793 (2.663,5.027)	40.234(28.433,53.323)	−2.369(−2.774,-1.963)	<0.001
Western Sub-Saharan Africa	22.335(5.637,86.213)	680.388(168.434,2642.939)	20.901 (6.166,62.637)	335.901(96.316,1015.996)	−2.249(−2.639,-1.858)	<0.001

At the SDI regional scale, the High SDI region had the highest number of LRIs deaths (69,216) due to non-optimal temperatures in 2019.The Low SDI region had the highest number of DALYs (3,178,353) and also had the highest ASMR and ASDR, at 5.182 (2.672,10.288) and 224.414, respectively (109.534,472.228). ASMR in the Middle SDI region and ASDR in the High SDI region were the lowest at 2.007 (1.325,2.999) and 44.959 (34.543,56.527), respectively. Between 1990 and 2019, non-optimal temperatures led to a decrease in ASMR and ASDR of LRIs in all five SDI regions showed a decreasing trend that was statistically significant. The Middle SDI region showed the largest decrease with AAPCs of −3.910 (−4.243, −3.576) and − 5.573 (−5.894, −5.251), respectively.

For the GBD region, the top three deaths were in South Asia, Western Europe, and Western Sub-Saharan Africa. Southern Latin America had the highest ASMR of 8.437 (5.507,11.923). The highest DALYs were in South Asia, Western Sub-Saharan.

Africa, North Africa and Middle East with 26.673 (13.211,41.393), 20.901 (6.166,62.637), 6.044 (4.145,8.039), and Western Sub-Saharan Africa has the highest ASDR of 335.901 (96.316,1015.996). Between 1990 and 2019, almost all of the 21 GBD regions show a decreasing trend in ASMR and ASDR, with the most pronounced decreasing trend in East Asia, where the AAPC are −5.582 (−6.343,- 4.185) and − 8.017 (−8.617,-7.413) respectively.

**Figure 2:**
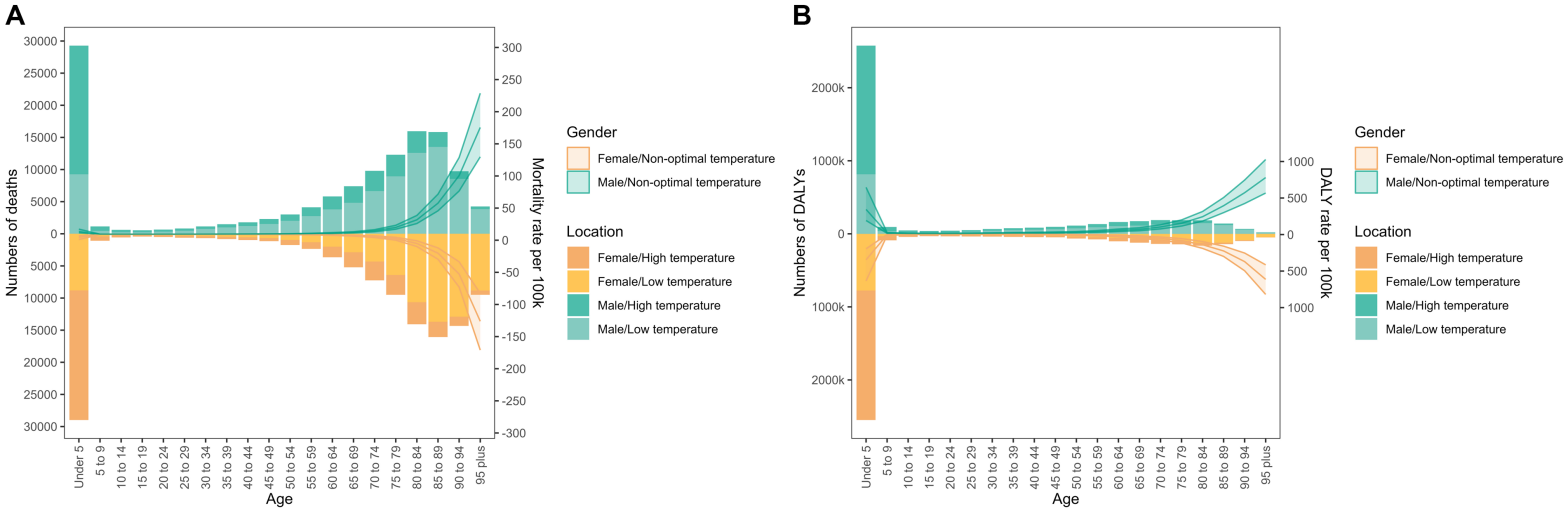
Age distribution of deaths (A) and disability-adjusted life years (B) in LRIs due to non-optimal temperatures. Bars show the number of deaths and disability-adjusted life years from lower respiratory infections due to non-optimal temperatures. The line at 95% UI represents the age-specific mortality and DALY rates due to non-optimal temperatures.

As shown in Figure 2, the overall trends for DALYs and deaths were similar. For both sexes, the majority of deaths from LRIs due to non-optimal temperatures occurred in the 0–5 and > 70 age groups. Globally, the burden of LRIs due to cold temperatures accounted for 64.33 and 52.04% of non-optimal temperature-induced LRI deaths and disability-adjusted life years, respectively. In addition, the burden is generally higher in males compared with females, with more deaths from lower respiratory tract infections in the age-specific group over 70 years.

### 3.2 Association of burden of LRIs due to non-optimal temperatures with SDIs

**Figure 3:**
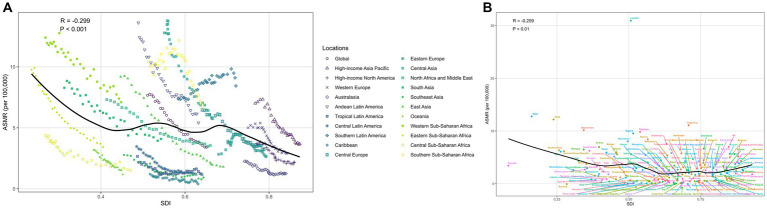
Correlation of SDI with LRIs mortality due to abnormal temperatures **(A)** global and 21 GBD regions; **(B)** 204 countries and territories.

Figure 3 and Supplementary Figure S2 shows the ASMR and ASDR for each GBD region and 204 states and territories for the period 1990–2019 plotted against the SDI for the same years, with the expected burden shown as a black line. Overall, a general downward trend in the expected relationship between SDI and non-optimal temperature-induced ASMR and ASDR is observed. At the regional level, ASMR and ASDR in Central Asia show a significant decreasing trend with increasing SDI. In Western Sub-Saharan Africa, Andean Latin America, Southern Sub-Saharan Africa, Southern Latin America, Central Asia, and High-income Asia Pacific, the observed ASMR estimates for LRIs are higher than expected based on the SDI in 1990–2019. ASDR estimates are higher than expected in Western Sub-Saharan Africa, Southern Sub-Saharan Africa, Central Asia, and High-income Asia Pacific. In 2019, as socio-economic growth in the region increases, ASDR estimates are higher than expected in Western Sub-Saharan Africa, Southern Sub-Saharan Africa, Southern Latin America, Central Asia, and High-income Asia Pacific. Asia Pacific. In 2019, the burden on LRI decreases as socioeconomic development and SDI increase. In many countries and regions, such as Niger, Chad, and Argentina, ASMR and ASDR are higher than expected.

### 3.3 Age-period-cohort modeling of the burden of LRIs

**Figure 4:**
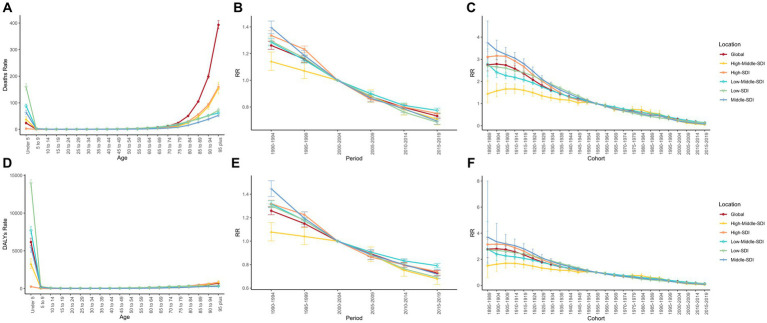
Age, period, and cohort effects of burden of LRIs, 1990–2019. **(A–C)** Age, period, and cohort effects of burden of deaths, respectively; **(D-F)** Age, period, and cohort effects of burden of DALYs, respectively.

The results of the age-cycle-cohort model, shown in Figure 4, indicate that the trends are broadly similar globally and across SDI regions. The burden of non-optimal temperature-induced LRIs (Deaths and DALYs) between the ages of 5 and 70 years was low overall, with the burden increasing with decreasing age in the under-five interval, with a significant risk of burden in the Low SDI region, which was much higher than the other regions, followed by the Low Middle SDI region. In contrast, the mortality rate in the 70+ zone shows an increasing trend with age, with a higher global mortality rate. Deaths Rate 95%CI for the under-five group was 23.821 (23.073, 24.594). Because of the increase in mortality in the 70+ interval, the Deaths Rate 95%CI for the 95+ group was as high as 159.650 (150.655, 169.182). Sex-stratified LRIs burden age-period-cohorts are presented in Supplementary Figures S3–S6. The highest mortality rates in the sex-specific trends were in the High Middle SDI and High SDI regions, respectively. The period effect showed a significant downward trend. The risk of death for LRIs declined in the five SDI regions and globally during this period. However, there was limited improvement in the risk of LRIs due to non-optimal temperatures in the High Middle SDI region from 1990 to 2004, particularly in men. In addition, there was an overall downward trend in cohort effects. Globally, the risk of death from LRIs and DALYs was greatest overall and for females born between 1900 and 1904, and for males for the cohort born between 1895 and 1899, with RR of cohort deaths of 2.787, 3.016, and 2.712, respectively, and of DALYs of 2.805, 3.041, and 2.752, respectively. Higher risk for Middle SDI and lower risk for High Middle SDI. Overall, a similar downward trend was observed in both the period effect and the cohort effect, with a more pronounced downward trend observed in the Middle and High SDI regions, where the risk of death in the period decreased from a decrease of 0.701, and the risk of DALYs decreased from 0.727. The risk of death in the cohort decreased from 3.745 to 0.074, and the risk of DALYs decreased from 3.702 to 0.075.

### 3.4 Projected trends in the burden of LRIs, 2020–2029

**Figure 5:**
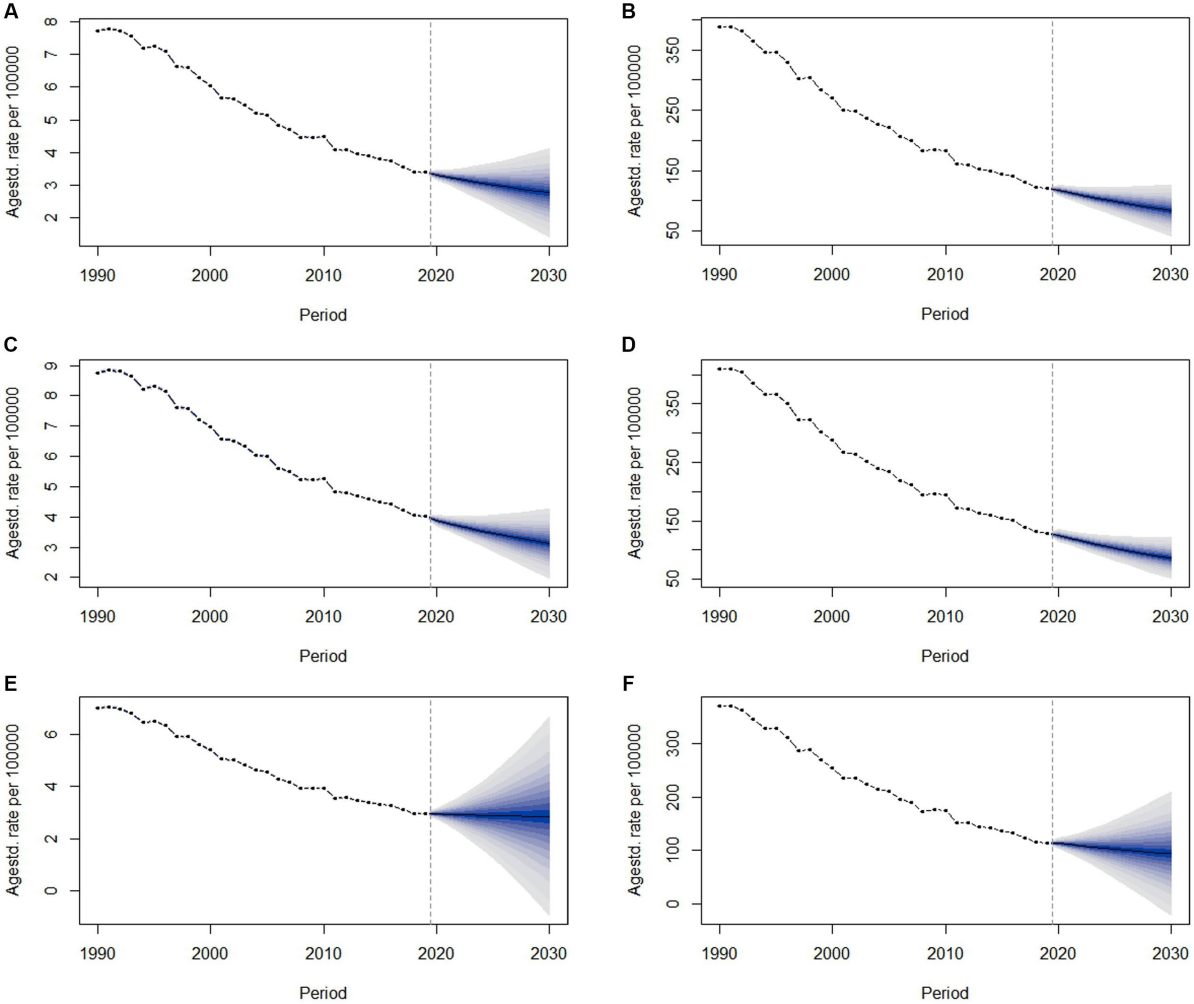
Observed and projected trends in the global burden of LRIs due to non-optimal temperatures from 1990 to 2030. **(A,B)** Observed and projected mortality and DLAY trends for all, **(C,D)** observed and projected mortality and DLAY trends for males, and **(E,F)** observed and projected mortality and DLAY trends for females.

Figure 5 shows the change in burden from 2020 to 2030 as predicted by the Bayesian Age-Period Cohort (BAPC) model, as well as the change in LRI burden by gender. The results in Supplementary Table S1 show that the model fit accuracies for the full population, male, and female projections are all above 99%, indicating that the model predictions are good. The curves fluctuate slightly, with similar trends for males and females, but with higher age-standardized mortality rates for males. The global age-standardized mortality and DALY rates for males are projected to decline gradually, to about 3.119/100,000 and 86.162/100,000 in 2030 according to the BAPC model, while for females the decline is more gradual, with mortality rates declining almost linearly, and are projected to fall to about 2.852/100,000 and 94.002/100,000 in 2030. For females, the decline is more moderate and almost linear and is projected to fall to about 2.852/100,000 and 94.002/100,000 in 2030.

## 4 Discussion

In this work, based on data from the GBD 2019 study, we provide up-to-date information on the burden of LRIs (deaths and disability-adjusted life years) due to non-optimal temperatures for 204 countries and territories from 1990 to 2019, providing a comprehensive picture of the current status and spatial and temporal patterns of the burden of LRIs due to non-optimal temperatures. Our findings indicate that in 2019, there were a total of 245.814 (174.76,342.302) thousand deaths and 85.108 (53.865,141.787) million disability-adjusted life years of LRIs due to non-optimal temperatures globally. Between 1990 and 2019, the APC model showed an overall decreasing trend for the period and cohort. The BAPC model predicts a downward trend from 2020 to 2030 as well, with a flat trend for women. Nevertheless, LRIs continue to pose a significant public health challenge, particularly in low socio-demographic index (SDI) areas, where higher age-standardized mortality rates (ASMR) and age-standardized disability rates (ASDR) are evident, consistent with the findings reported by Kang et al. (3).

Furthermore, LRIs contribute to a significant number of deaths in children under 5 years of age and adults over 70 years of age. Our findings call for targeted and sustained efforts to reduce the burden of LRIs due to unsuitable temperatures.

Prior studies have generally found that the burden due to low temperatures is significantly higher than that due to high temperatures. For example, the GBD 2019 First Estimates and Published Study on Non-Optimal Temperature Exposure and the Burden of Risk for All-Cause Mortality indicates (27) that the burden due to low temperatures is 2.2 times greater than the burden due to high temperatures. Among other things, LRIs are more sensitive to nonoptimal temperatures compared with other diseases. In addition, a report on the global burden of lower respiratory tract infections showed the proportion of DALYs in each GBD region. Among them, the highest percentage of DALY due to low temperature was found in Western Europer (21%).The highest percentage of DALY due to high temperature was found in South Asia and Western Sub-Saharan Africa (7.8%) (28).The present study may reveal regional differences in the burden of lower respiratory infections due to non-optimal temperatures on a global scale. Different climates at different longitudes and latitudes may result in different levels of disease burden. Studies in Canada, Finland, and China have consistently shown that cold is associated with an increased risk of morbidity, exacerbation, mortality, and hospitalization from lower respiratory tract infections, especially for children and the older adult (29–31). The underlying mechanism may be that temperature has a significant effect on viral activity, exacerbating viral infections leading to lower respiratory tract infection burden (32, 33). Although the results showed that almost all GBD regions had a higher ASMR and ASDR decreased, there is still a need for continued attention. Consistent with the World Health Organization report, our study showed that the burden of LRIs was highest in low SDI regions, Africa, in 2019 (34). The results of the analysis of the association between the burden of LRIs due to non-optimal temperatures and SDI showed an overall decreasing trend in burden with higher SDI. Higher burden in low socioeconomic status areas may be associated with incomplete access to health care and lack of resources. Therefore, prevention of the occurrence of LRIs and intensification of treatment should be sustained, focusing on multisectoral actions to reduce the burden in these areas. Notably, LRI deaths due to non-optimal temperatures increased in high SDI areas from 1990 to 2019, mainly due to higher ASMR in the older adult population. And the aging population in high SDI areas continues to rise. In addition, in low-income countries, the low heating rates and widespread use of biomass fuels significantly increase exposure to wood smoke, which is associated with higher rates of respiratory infections. In contrast, in high-income regions, the use of effective heating systems and high-efficiency particulate filters greatly improves indoor air quality and reduces pathogen concentrations, thereby lowering the risk of lower respiratory infections. This may explain the differences in lower respiratory infection rates between the two regions.

Finding susceptible populations is more beneficial in coping with the burden of lower respiratory infections caused by non-optimal temperatures. Because of the intricate interactions between age, period, and cohort factors, we applied age-time-cohort modeling and the IE algorithm to quantify their net effect on the burden of LRIs. Our findings suggest that most deaths occur in the <5 and > 70 years age groups, and APC modeling shows that the burden of LRIs due to non-optimal temperatures (mortality and DALY rates) increases globally with age in the >70 years age group. The burden is highest in low SDI areas in the <5 years age group, especially ASDR. infants, young children, and the older adult are more vulnerable to temperature changes due to underdeveloped or impaired respiratory and thermoregulatory capacities (35, 36). Therefore, the ongoing promotion of children’s health and the reduction of the burden of LRIs in children under 5 years of age need to be accompanied by an attention to the increase in the burden of LRIs due to the aging of the population, especially in high SDI areas. This finding suggests that equal attention should be given to children and older adults to address the burden of LRIs due to non-optimal temperatures. The APC model decomposes the burden information across regions into age, period, and cohort effects. Period effects are changes in medical technology, economic development, and disease classification criteria during a given study period, and the RR for period effects in this study was a continuous decline. The cohort effect represents the long-term trend in the burden of death for people born in the same time period and represents the influence of early socioeconomic, behavioral, and environmental factors on the risk of lower respiratory tract infections. The RR for the cohort effect has a high value domain in the early birth cohort and shows a continuous decreasing trend to the most recent birth cohort as a whole. This is a result of global socioeconomic development, prevention of environmental risks, medical advances, and increased focus on prevention of lower respiratory tract infections. Risks associated with periods and cohorts are decreasing over time, but there is also the problem of uneven improvement in different regions.

Forecasting trends in the burden of lower respiratory infections helps to understand the burden of disease and assists in public health resource allocation decisions. Based on the predictions of the BAPC model in the study, a gradual decline in global age-standardized death and DALY rates is expected in 2030. In terms of trends, there are some gender differences. Males have a high initial burden but a clear downward trend. The projected burden of disease for females is stabilizing, and therefore, a comprehensive strategy of sustained attention to the non-optimal temperature burden of lower respiratory tract infections in females is needed to reduce the burden of disease and achieve better health outcomes for people with LRIs.

The GBD study used in this study is one of the most authoritative databases, using a harmonized and standardized approach to compensate for scarce and biased data on the actual burden of disease. Up-to-date information on the burden of lower respiratory tract infections due to non-optimal temperatures at global, regional and national levels is provided. This study still has some limitations. First, the GBD database has only clinical diagnoses and lacks further laboratory or imaging evaluations, which may lead to recall bias (37).Second, estimates of non-optimal temperatures are time- and space-dependent, and it is difficult to account for both factors at the same time (38). The indicator of the burden of LRIs due to non-optimal temperatures is only an estimate, and there is a lack of comprehensive national data to take into account the impact of modern facilities on lower respiratory tract infections, so the effect of nonoptimal temperatures on LRIs may be underestimated. Finally, the prediction based on the BAPC model did not take into account the occurrence of COVID-19, which may be somewhat different from reality.

## 5 Conclusion

Our study comprehensively elucidates the distribution and dynamic trends in the burden of lower respiratory tract infections attributable to non-optimal temperatures from 1990 to 2019 across multiple dimensions. While the overall burden of deaths and disability-adjusted life years (DALYs) exhibited a decreasing trend, this improvement was uneven across different regions. Furthermore, the results indicate that targeted efforts should be made to mitigate lower respiratory health losses among infants, young children, and the older adult. To effectively reduce the burden of lower respiratory tract infections, sustained global public health policies and interventions are essential.

## Data Availability

The datasets presented in this study can be found in online repositories. The names of the repository/repositories and accession number(s) can be found at: https://vizhub.healthdata.org/gbd-results/.

## Footnotes

^1^ https://analysistools.cancer.gov/apc/
