# Supplementary material for: Systematic analysis and prediction of the burden of lower respiratory tract infections attribute to non-optimal temperature, 1990–2019
Source: Front Public Health. 2024 Oct 18;12:1424657. doi: 10.3389/fpubh.2024.1424657 (PMC11530990; doi:10.3389/fpubh.2024.1424657)
Supplement: Supplementary file 1 [file Data_Sheet_1.docx]

| A  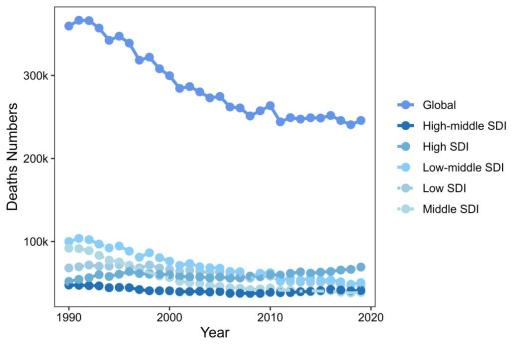 | B  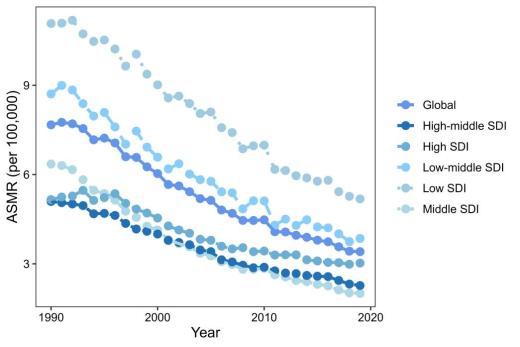 |
| --- | --- |
| C  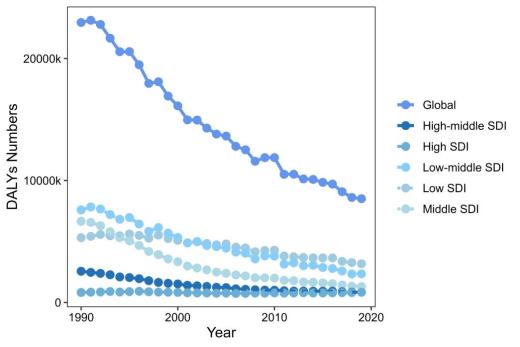 | D  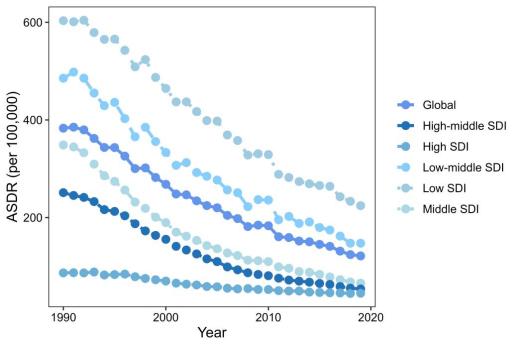 |

**Supplementary FIGURE1**

Burden of LRIs caused by non-optimal temperatures, 1990-2019, (A) number of deaths (B) ASMR (C) number of DALYs (D) ASDR

| A  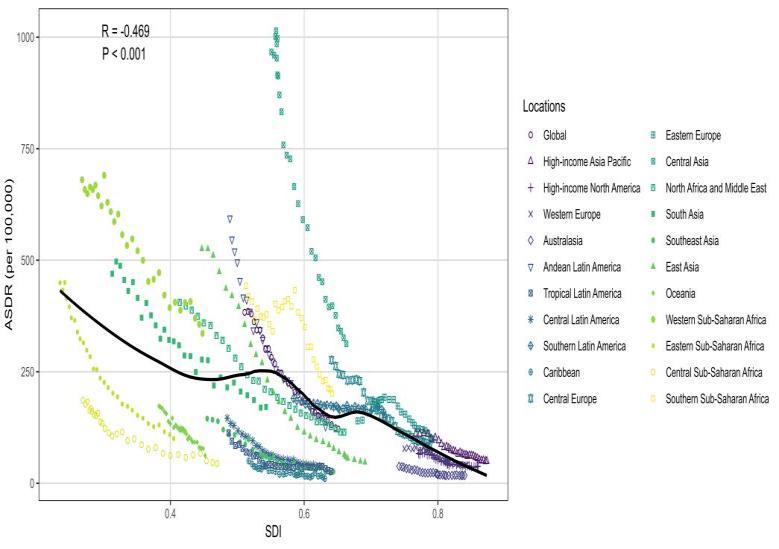 | B  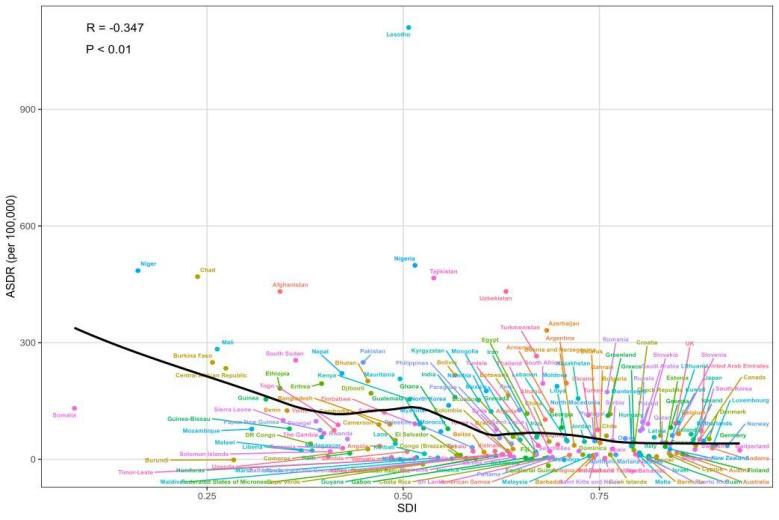 |
| --- | --- |

**Supplementary FIGURE2**

Correlation of SDI with rates of DALYs in LRIs due to non-normal temperatures (a) global and 21 GBD regions; (b) 204 countries and territories

| A  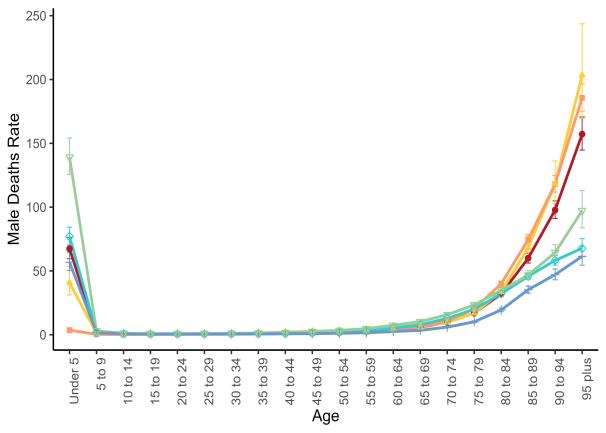 | B  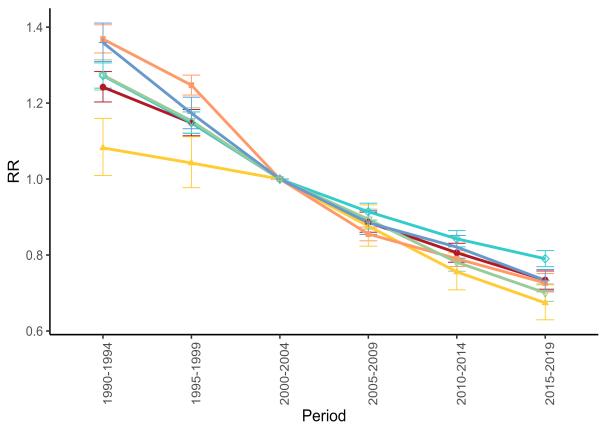 | C  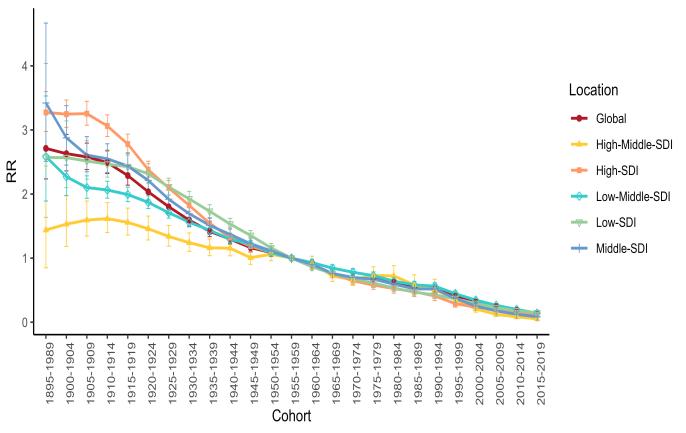 |
| --- | --- | --- |

**Supplementary FIGURE 3**

Age, period, and cohort effects of the burden of death in male with LRIs, 1990-2019. (A-C) Age, period, and cohort effects, respectively;

| A  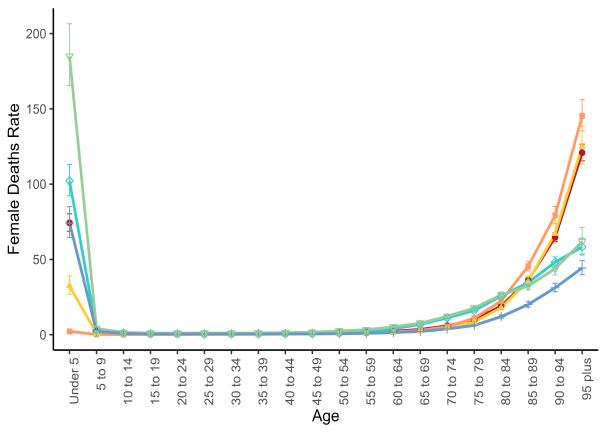 | B  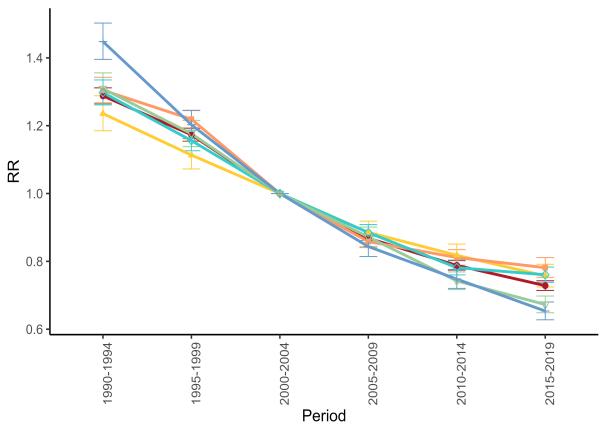 | C  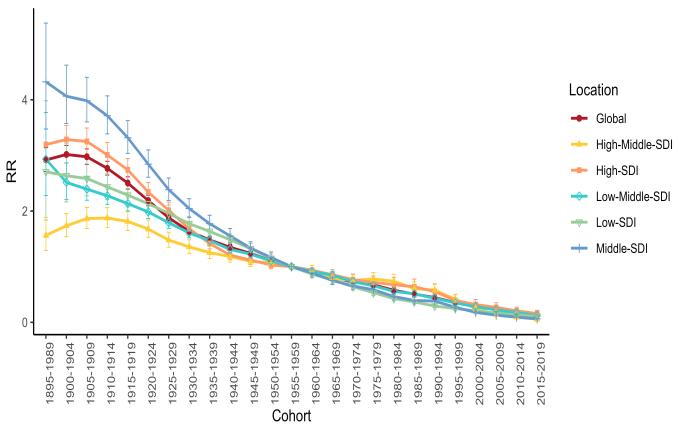 |
| --- | --- | --- |

**Supplementary FIGURE 4**

Age, period, and cohort effects of the burden of death in Female with LRIs, 1990-2019. (A-C) Age, period, and cohort effects, respectively;

| A  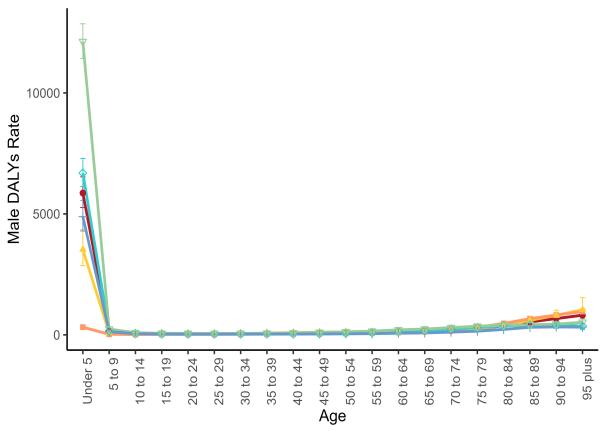 | B  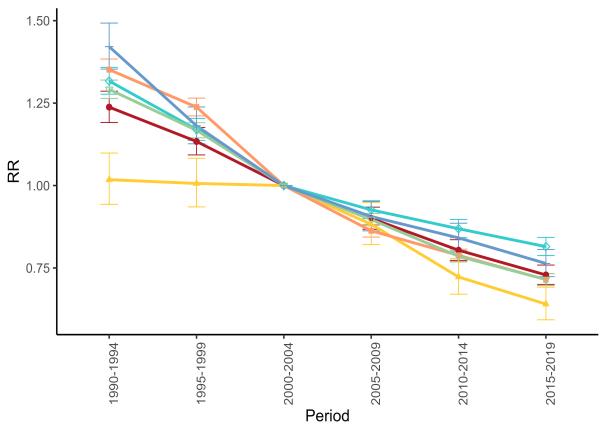 | C  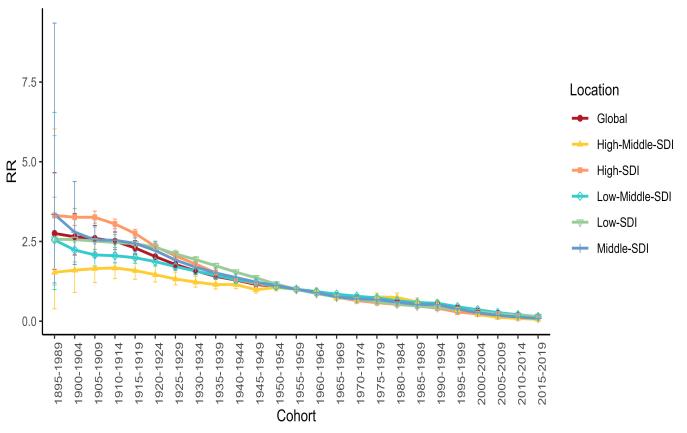 |
| --- | --- | --- |

**Supplementary FIGURE 5**

Age, period, and cohort effects of the burden of DALYs in Male with LRIs, 1990-2019. (A-C) Age, period, and cohort effects, respectively;

| A  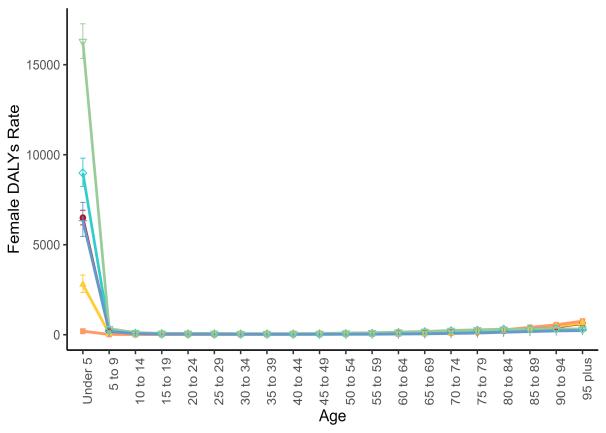 | B  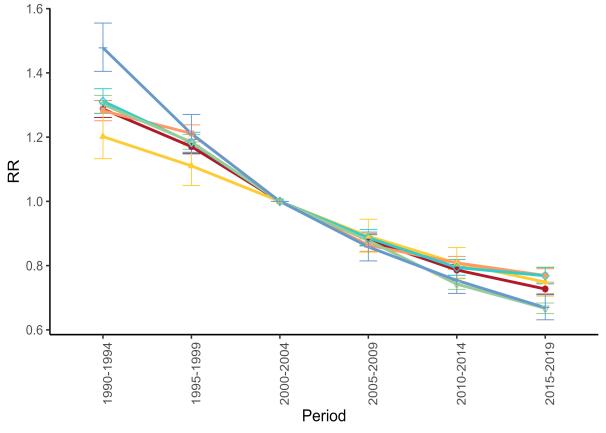 | C  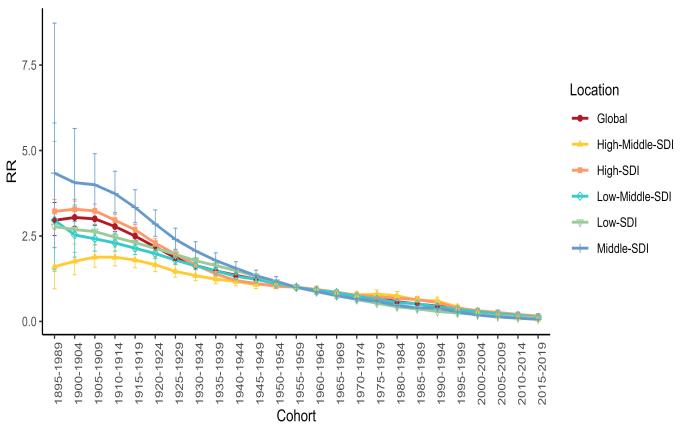 |
| --- | --- | --- |

**Supplementary FIGURE 6**

Age, period, and cohort effects of the burden of DALYs in Female with LRIs, 1990-2019. (A-C) Age, period, and cohort effects, respectively;

**Supplementary Table 1**

Indicators associated with predictive model fits for the burden of disease from lower respiratory tract

infections due to non-optimal temperatures, 1990-2019

| Variables | MSE | MAE | MAPE(%) | Fitting accuracy(%) |
| --- | --- | --- | --- | --- |
| Standardized mortality rate | 0.0006 | 0.0196 | 0.3354 | 99.6646 |
| Standardized mortality rate（Male） | 0.0007 | 0.0231 | 0.3426 | 99.6574 |
| Standardized mortality rate（Female） | 0.0004 | 0.0171 | 0.3339 | 99.6661 |
| Standardized DALYs rate | 3.3086 | 1.4926 | 0.5878 | 99.4121 |
| Standardized DALYs rate（Male） | 4.3584 | 1.6991 | 0.6181 | 99.3818 |
| Standardized DALYs rate（Female） | 2.4544 | 1.2993 | 0.5536 | 99.4464 |
